# Forming Bonds While Breaking Old Ones: Isomer-Dependent Formation of H_3_O^+^ from Aminobenzoic Acid During X-ray-Induced Fragmentation

**DOI:** 10.1021/acs.jpca.2c06869

**Published:** 2023-02-07

**Authors:** Abdul Rahman Abid, Onni Veteläinen, Nacer Boudjemia, Eetu Pelimanni, Antti Kivimäki, Matti Alatalo, Marko Huttula, Olle Björneholm, Minna Patanen

**Affiliations:** † Nano and Molecular Systems Research Unit, University of Oulu, 90570 Oulu, Finland; ‡ Molecular and Condensed Matter Physics, Uppsala University, 75120 Uppsala, Sweden; § MAX IV Laboratory, Lund University, 22100 Lund, Sweden

## Abstract

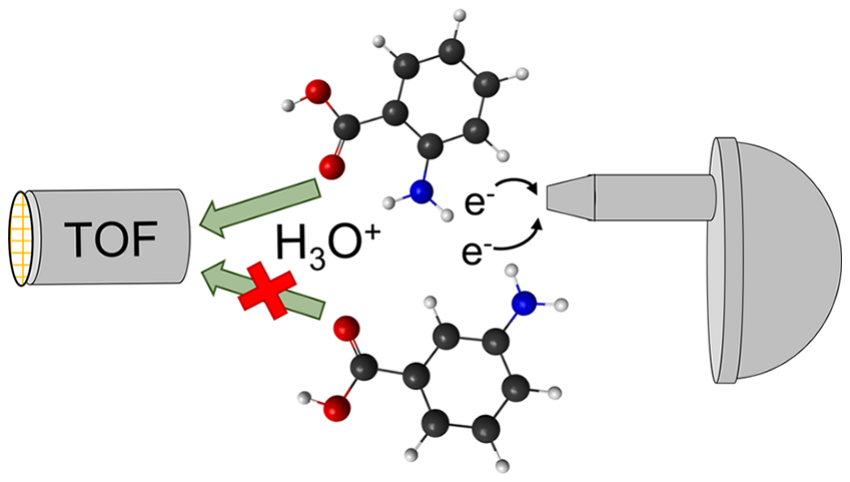

Intramolecular hydrogen transfer, a reaction where donor and acceptor sites of a hydrogen atom are part of the same molecule, is a ubiquitous reaction in biochemistry and organic synthesis. In this work, we report hydronium ion (H_3_O^+^) production from aminobenzoic acid (ABA) after core-level ionization with soft X-ray synchrotron radiation. The formation of H_3_O^+^ during the fragmentation requires that at least two hydrogen atoms migrate to one of the oxygen atoms within the molecule. The comparison of two structural isomers, ortho- and meta-ABA, revealed that the production of H_3_O^+^ depends strongly on the structure of the molecule, the ortho-isomer being much more prone to produce H_3_O^+^. The isomer-dependency suggests that the amine group acts as a donor in the hydrogen transfer process. In the case of ortho-ABA, detailed H_3_O^+^ production pathways were investigated using photoelectron-photoion-photoion coincidence (PEPIPICO) spectroscopy. It was found that H_3_O^+^ can result from a direct two-body dissociation but also from sequential fragmentation processes.

## Introduction

Intramolecular hydrogen transfer is a process where a hydrogen atom moves within a molecule and attaches to a new site. It can induce fragmentation or decomposition of the molecule and plays a significant role in organic synthesis and various reactions of biomolecules.^1−3^ In combustion processes, it is considered to take place in ringlike transition states.^3^ In many biological compounds, intramolecular hydrogen transfer is initiated by radiation or oxidative damage and involves different sites such as thiol, amine, and hydroxyl groups as well as backbone carbons.^4^ Since intramolecular hydrogen transfer is crucial in many processes, an improved understanding of its effects and conditions in various molecules is of utmost importance. Single-molecule gas-phase studies offer a platform for accurate experimental observations which can be compared directly to high-level theoretical modeling. Recently, multicoincidence experiments, where several charged particles (electrons and/or ions) originating from the same ionization event are detected, have been used to get insight into gas-phase fragmentation mechanisms involving transfer of at least two hydrogens to an atom which in the molecular ground state is several bonds apart. In these experiments, the nuclear dynamics involving hydrogen transfer are initiated by bringing the molecule to a singly or doubly ionized state via, for example, laser,^5^ ion,^6^ and extreme ultraviolet^7,8^ or X-ray^9^ irradiation.

**Figure 1:**
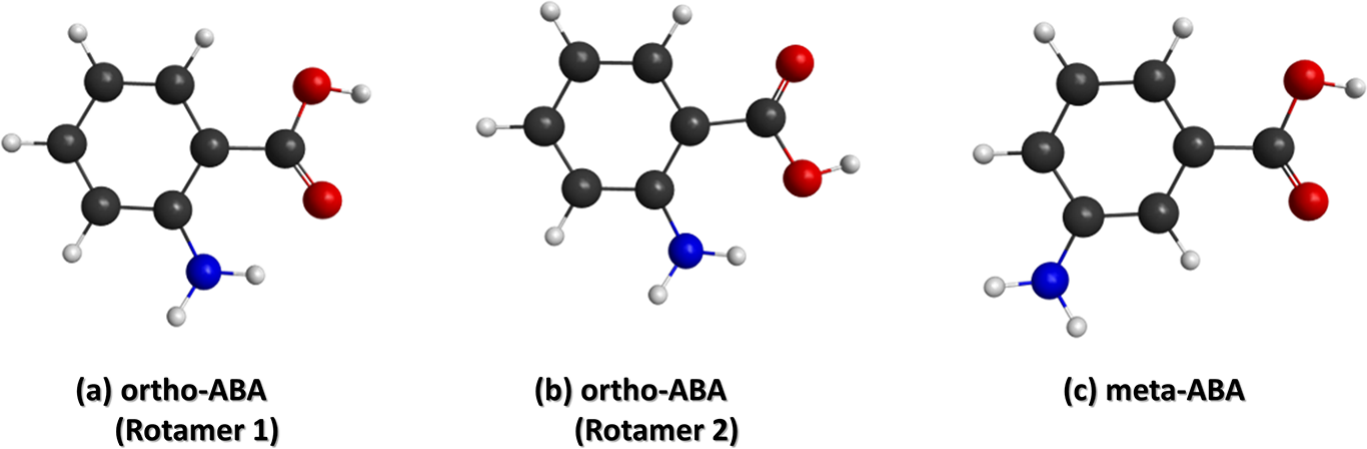
Optimized geometries of aminobenzoic acid isomers. According to our calculations, rotamer 1 has about 120 meV lower ground state energy than rotamer 2 and is thus mostly populated in this gas-phase experiment. Black, red, blue, and gray balls depict carbon, oxygen, nitrogen, and hydrogen atoms, respectively.

In this work, we have used synchrotron radiation to induce core ionization in two isomers of gas-phase aminobenzoic acid (ABA): ortho-aminobenzoic acid (ortho-ABA) and meta-aminobenzoic acid (meta-ABA). Figure 1 presents the structural formulas of ABA isomers; for the ortho-isomer, two rotamers, which differ by a 180° rotation of the carboxylic functional group,^10^ are shown. After core ionization, the molecule is in a highly energetic state and decays within a few femtoseconds via Auger decay to doubly charged final states, many of which are dissociative leading to the fragmentation of the molecule. The fragmentation process implies bond-breaking, but nuclear dynamics and rearrangements may also lead to the formation of new bonds. We especially concentrate on the formation of a hydronium ion (H_3_O^+^), which requires at least two hydrogens to be dislocated from their initial positions in the neutral ground state. As we only detect the final product, singly charged hydronium ion, we do not have information on the charge of the migrating hydrogens (neutral atoms or protons). We refer to the process generally as hydrogen transfer since transfer of two or more protons would require consequently electron transfer to lead to the observed charge state.

H_3_O^+^ is a protonated form of water taking part in many terrestrial and interstellar chemical processes.^11−14^ In aqueous solutions, proton transfer and hopping mechanisms via H_3_O^+^ formation have been studied for a long time, but there are still caveats in the detailed understanding of the dynamics.^15,16^ Gas-phase H_3_O^+^ is found in interstellar media where its formation is explained by intermolecular hydrogen/proton transfer reactions.^12^ H_3_O^+^ formation via intermolecular H transfer has also been observed in water dimers by electron–ion coincidence spectroscopy^17^ and in larger water clusters after core ionization.^18^ Several mass spectrometric studies have reported H_3_O^+^ formation in alcohol molecules by intramolecular hydrogen migration and decomposition of metastable intermediate ions, and theoretical calculations have elucidated these possible pathways.^19−22^ Recently, mechanisms for single and double hydrogen migration in ethanol leading to the formation of H_2_O^+^ and H_3_O^+^ were studied by Kling et al.^5^ The combination of femtosecond laser pump–probe experiments and *ab initio* molecular dynamics simulations revealed that the two hydrogens participating in H_3_O^+^ production are not correlated, and they have no preferred sites of origin in the molecule. It was shown that the transfer of the second hydrogen is concerted with the breakage of the C–O bond. The time scales of H transfer processes were found to depend strongly on the internal energy in the system, varying from tens of femtoseconds for the first H transfer up to hundreds of femtoseconds for the second transfer. Castrovilli et al. found experimentally a single hydrogen transfer time of 48 fs for the amine-to-carboxyl H transfer in glycine,^8^ in agreement with *ab initio* molecular dynamics simulations.^6^

In this work, we show that H_3_O^+^ production from ABA is very sensitive to the differences in the relative positioning of functional groups between the different isomers. We shed light on the production pathways with photoelectron-photoion-photoion coincidence (PEPIPICO) spectroscopy. We have found a direct two-body dissociation channel which can be followed by a further decay of the heavy cofragment. A mechanism, where a neutral CO is emitted first, is also discussed.

## Experimental Methods

The experiment was carried out at the gas-phase endstation^23^ of the FinEstBeAMS beamline^24^ at the MAX IV Laboratory, Sweden, similarly to a recently reported PEPIPICO study of photofragmentation of avobenzone.^25^ A detailed description of the experimental setup can be found in ref (23), and only a short description is given here. Powder-form samples (purity of >98% from Sigma-Aldrich, Merck Group, St. Louis, US) were placed in a resistively heated oven in a stainless steel crucible. Effusive beams of gas-phase samples were created by heating the samples to ∼60 °C (ortho-ABA) and ∼120 °C (meta-ABA). The sample vapor was intersected by X-rays in the initially field-free interaction region between a hemispherical electron energy analyzer and an ion time-of-flight spectrometer. The electron analyzer (Scienta R4000, Scienta Omicron GmbH) is equipped with a fast position-sensitive detector (Quantar Inc., Model 3395A). The electron detection within the kinetic energy range of interest triggered a ramp-up of an electric field in the interaction region. Positive ions originating from the same ionization event were thus extracted toward a modified Wiley–McLaren-type multihit-capable ion TOF spectrometer equipped with a position-sensitive delay line detector (HEX80, RoentDek Handels GmbH). In the same measurement, the extraction field was also triggered by a random pulse generator in order to model and subtract “false” or random coincidences from the coincidence data.^26^ The amount of false coincidences was kept low by limiting the electron count rate to 10–20 counts/s. Ions were detected in coincidence with photoelectrons from the C 1s, N 1s, and O 1s core levels. The photon energy was selected to be approximately 50–60 eV above the corresponding core ionization threshold, so the photon energies 350, 450, and 600 eV were used for C 1s, N 1s, and O 1s photoionization, respectively. The electron analyzer was operated with a pass energy of 100 eV and curved entrance slit of 0.8 mm, and photoelectrons were collected in about a 10 eV wide kinetic energy window. The C 1s photoelectron spectrum was calibrated with respect to the C 1s photoelectron line of CO_2_ with vertical ionization energy of 297.699 ± 0.03 eV.^27^

## Results and Discussion

### Differences in H_3_O^+^ Production between Ortho and Meta Isomers

**Figure 2:**
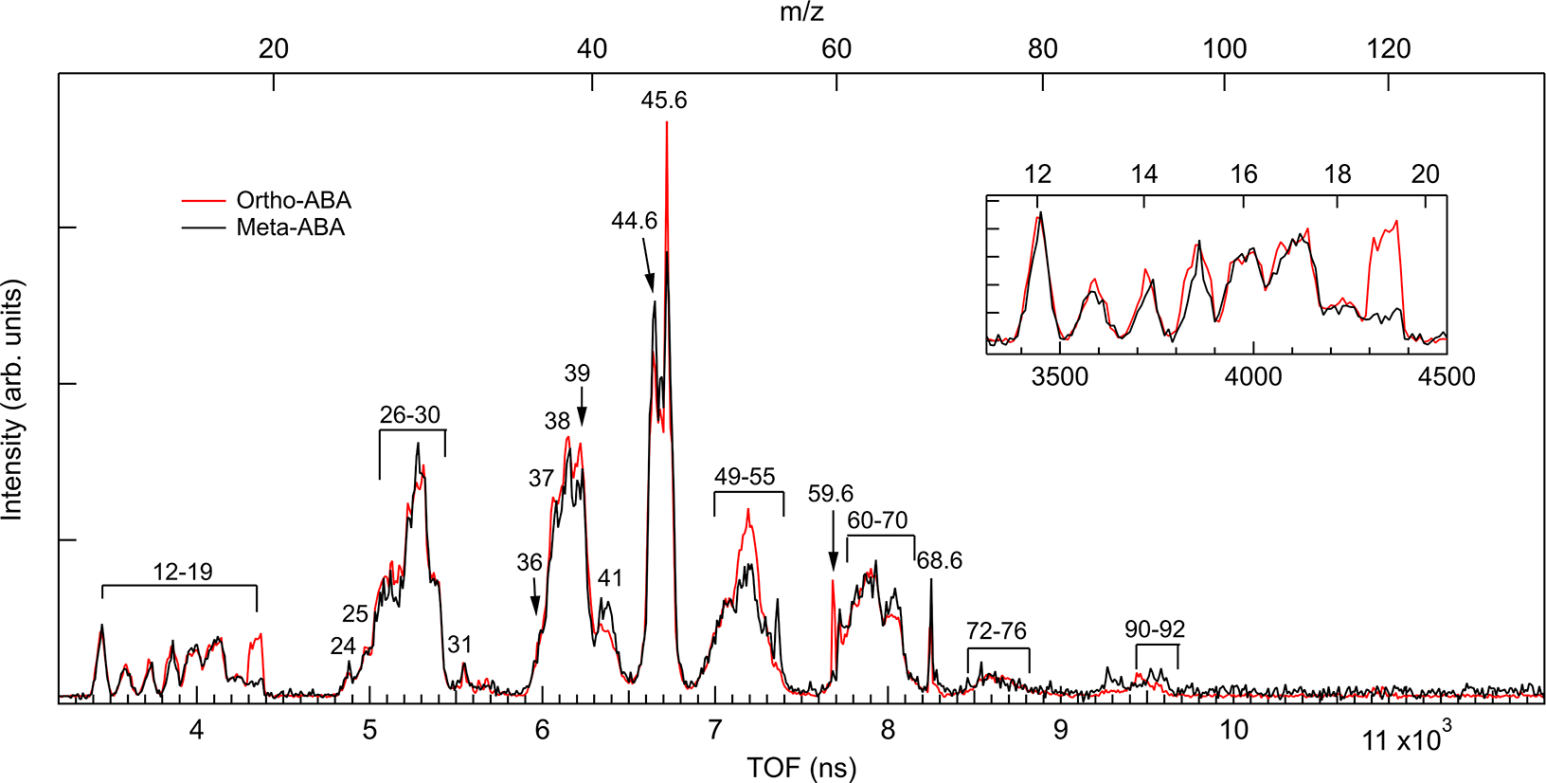
Comparison of the C 1s coincident TOF spectra of ortho- (red line) and meta-ABA (black line) recorded at 350 eV photon energy. The top axis indicates the corresponding *m*/*z* range. Inset: enlarged spectra corresponding to the *m*/*z* range of 11–20 normalized with respect to the *m*/*z* = 12 peak.

Comparison of time-of-flight (TOF) mass spectra recorded in coincidence with C 1s photoelectrons for ortho- and meta-ABA is presented in Figure 2. The *m*/*z* scale is sketched on the top axis, and *m*/*z* values are given for most abundant fragments. For an easier visual comparison between the isomers, the spectra have been normalized to have equal integrated intensity in the TOF range corresponding to a *m*/*z* range of 10–118. The mass spectra show a large number of peaks with different shapes, and both isomers have overall similar fragmentation patterns. Sharp peaks at noninteger *m*/*z* values are due to stable dications. A detailed breakdown of the mass spectra with possible fragments is presented in Table S4. The majority of the fragments consist of two or more C, N, or O atoms, plus some hydrogens. There are also ions containing only one C, N, or O atom, plus some hydrogens, as shown in the inset of Figure 2, presenting the zoom of the *m*/*z* = 11–20 region normalized to the *m*/*z* = 12 peak (C^+^).

The ion distribution is very similar for both ABA isomers, except for H_3_O^+^ (*m*/*z* = 19). This is not a fragment created by simple bond breaking, but its formation requires the motion of several atoms to form new bonds during the fragmentation process. In principle, *m*/*z* = 19 could also correspond to C_3_H_2_^2+^, which is a stable dication observed in several studies before (see, e.g., refs (28 and 29)). However, as will be shown later, this ion belongs to coincidence ion pairs whose mass would be larger than the parent molecule or would lead to a mass loss that cannot be explained by elements present in ABA, if assumed to be a doubly charged fragment with mass of 38 amu. Thus, *m*/*z* = 19 is interpreted to be mostly H_3_O^+^. The relative production of H_3_O^+^ is suppressed by 70% in meta-ABA compared to ortho-ABA as obtained from the integrated intensity of the *m*/*z* = 19 peak. The sensitivity to the distance between the NH_2_ and COOH functional groups indicates that amine hydrogen(s) are involved in the hydronium ion formation; the NH_2_ functional group is further away from the oxygen atom in meta-ABA than in ortho-ABA. A study by Seo et al.^30^ addressed a preferred site of proton attachment of gas-phase ABA isomers in neutral form and discussed the importance of resonance structures stabilizing the protonated isomers. In their infrared spectroscopy study, they observed N-protonated meta-ABA, whereas the spectrum of protonated ortho-ABA corresponded best with a modeled spectrum of a molecule with hydrogen shared between carboxyl and amine groups. Neutral ortho-ABA is expected to have a resonance structure, in which electron density from the amino group’s lone pair electrons is donated to the benzene ring and further to the carboxylic group, giving a net negative charge to the carboxylic group. Such a resonance is not possible in meta-ABA. Even if we must be careful when drawing parallels between our study, where most of the dynamics are expected to take place in the dicationic state, and studies of protonation of neutral molecules, the resonance structure in ortho-ABA may favor H/H^+^ transfer to the carboxyl group also here.

When looking at the neutral ground state geometry, if the hydrogen is transferred to the oxygen in the hydroxyl group, double hydrogen transfer is needed, whereas triple hydrogen transfer would be needed if the oxygen originates from the C=O group. Overall, the H_3_O^+^ contribution to the whole fragmentation is easily detectable; approximately 2% of the C 1s coincident ions are H_3_O^+^. Among the ions containing only one C, N, or O atom, however, it is quite prominent for ortho-ABA. The abundance estimate has not been corrected for the possibly slightly varying detection efficiency as a function of *m*/*z*. It should be also noted that H^+^ ions are not detected in our experiment due to the strong noise in the ion signals caused by the rising edge of the pulsed ion extraction field.

The TOF spectra recorded in coincidence with N 1s and O 1s photoelectrons (shown in Figure S1) show that the relative abundance of hydronium ions compared to total amount of ions decreases when going from C 1s photoionization to N 1s and O 1s. Despite the lower statistics of N 1s and O 1s acquisition, it can be estimated that the relative abundance of H_3_O^+^ is approximately 40–50% lower in the case of N 1s and O 1s coincidences compared to C 1s. This reduction in hydronium ion production can be caused by two factors: initial dynamics in the core ionized states and different population of final Auger states. Bond elongation during the lifetime of a core-ionized state (typically 3–6 fs for C, N, and O 1s ionized states^31^) can influence how the nuclear dynamics evolve after the Auger decay. On the other hand, there are many different dicationic states with different potential energy surfaces and their relative population via Auger decay changes depending on the site of core ionization. The Auger decay in molecules is to a first approximation a local process (so-called “one-center” approximation^32,33^), and direct Auger decay produces preferentially final states with configurations where the core ionized atom is doubly charged initially. Both above-mentioned effects can in principle drive the initial dynamics away from hydrogen transfer routes, but as in our experiment, we observe only the first photoelectron immediately after ionization and the final charged fragments microseconds after their creation and cannot obtain detailed information about these intermediate dynamics.

### Routes for H_3_O^+^ Formation in Ortho-ABA

**Figure 3:**
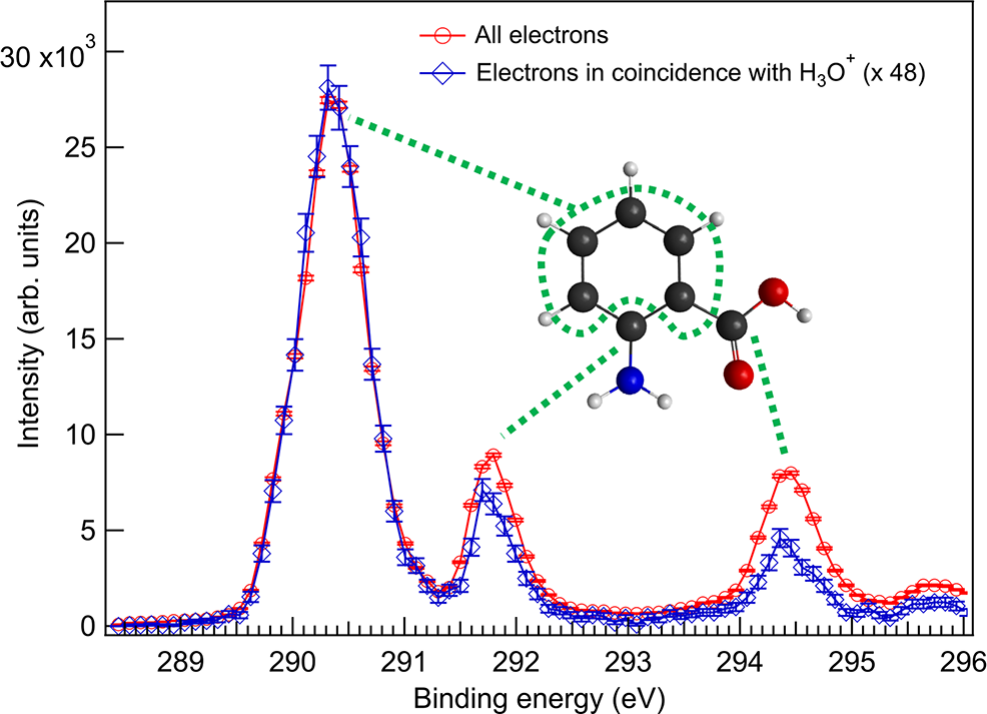
C 1s photoelectron spectrum of ortho-ABA without fragment filtering (red line with circles) and in coincidence with *m*/*z* = 19 (H_3_O^+^) fragment (blue line with diamonds). Dashed green lines indicate the sites from where the observed XPS peaks originate.

The PEPICO measurement allows looking at an ion-filtered photoelectron spectrum, i.e., filtering the electron data so that an electron spectrum represents only electrons in coincidence with a specific ion. A comparison between a total C 1s photoelectron spectrum and a spectrum of electrons in coincidence with *m*/*z* = 19 fragments (H_3_O^+^) is presented in Figure 3. The error bars in the photoelectron spectra represent statistical errors (square root of electron counts in each bin). The spectra consist of three well-resolved peaks, and to interpret them, molecular orbital energies from theoretical calculations were used (see the Supporting Information for computational details). The largest peak at 290.3 ± 0.05 eV corresponds to ionization of five carbon atoms in the benzene ring bound to other C’s. The two smaller ones of approximately equal intensity at 291.7 ± 0.05 eV and 294.4 ± 0.05 eV correspond to ionization of C’s in the C-NH_2_ and COOH groups, respectively. Compared to the total photoelectron spectrum, the H_3_O^+^ filtered photoelectron spectrum has clearly lower relative intensity in the COOH peak. Also, the relative intensity of the C-NH_2_ groups is slightly smaller. This observation is in line with the reduced H_3_O^+^ production when N 1s and O 1s are ionized: when the initial charge is on the C-NH_2_ or COOH groups, the H_3_O^+^ production is suppressed. Some additional insight into the H_3_O^+^ production could have been obtained by studying the coincidences between ions and Auger electrons, but due to limitations of available synchrotron measurement time, this is left for a future study. An Auger spectrum represents the binding energies of the dicationic states and is thus related to the internal energy of a molecular system. Auger electron-ion coincidences could have revealed if there is a trend in the H_3_O^+^ production as a function of internal energy. For a multiatomic molecular system, the Auger spectrum typically consists of many overlapping states, and interpretation of the fragmentation pattern in relation to specific final states is not straightforward. Inhester et al.^34^ studied the fragmentation of a prototypical “ESCA” molecule, CF_3_COOCH_2_CH_3_. They carried out a bond order analysis of a specific bond wrt. the final Auger state and showed that even at similar internal energies (dicationic final state binding energies) many electronic states of different bond characteristics are populated and give rise to different fragmentation patterns.

**Figure 4:**
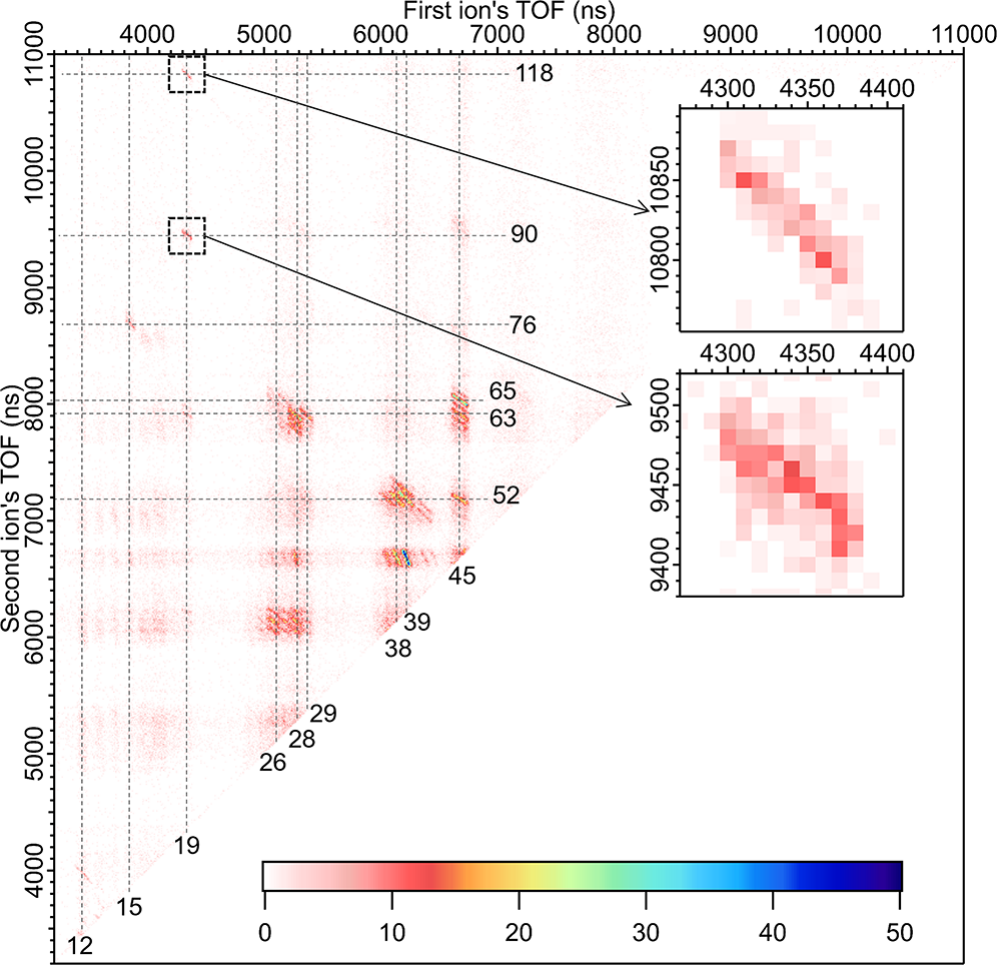
C 1s PEPIPICO 2D map of ortho-ABA. Black dashed boxes with zoom maps on the right side indicate the clearest patterns involving the H_3_O^+^ ion [ion pairs (19, 118) and (19, 90)].

Additional insight into the formation of H_3_O^+^ after C 1s photoionization can be obtained by looking at the two-dimensional PEPIPICO map presented in Figure 4. It is formed by plotting the TOF of the later arriving (heavier) ion as a function of the TOF of the first ion. The cation pairs originating from the same ionization event form “islands”, some of which have a clear slope. This is an indication of momentum correlation between the cations, and a comparison between the experimental and modeled slope (*k*_exp_ and *k*_cal_, respectively) can be used to evaluate a possible fragmentation mechanism through which the cations were formed.^35−38^ Overall, among the most intense coincidence patterns there are pairs with a fragment of the aminobenzene moiety (e.g., *m*/*z* = 38, 52, 63–65) and a fragment of the carboxyl end (*m*/*z* = 28–29, 45). An example of a prominent pair is (45, 65) which can be CHO_2_^+^ in coincidence with either C_5_H_5_^+^ or C_4_H_3_N^+^. Also, coincidence pairs with two charged fragments from the aminobenzene moiety are abundant, such as (38–39, 52). There are many ways how these *m*/*z* values can be obtained from the aminobenzene moiety depending on whether the lighter or heavier fragment carries the nitrogen. A full list of observed fragments and their suggested assignment is given in Table S4.

**Figure 5:**
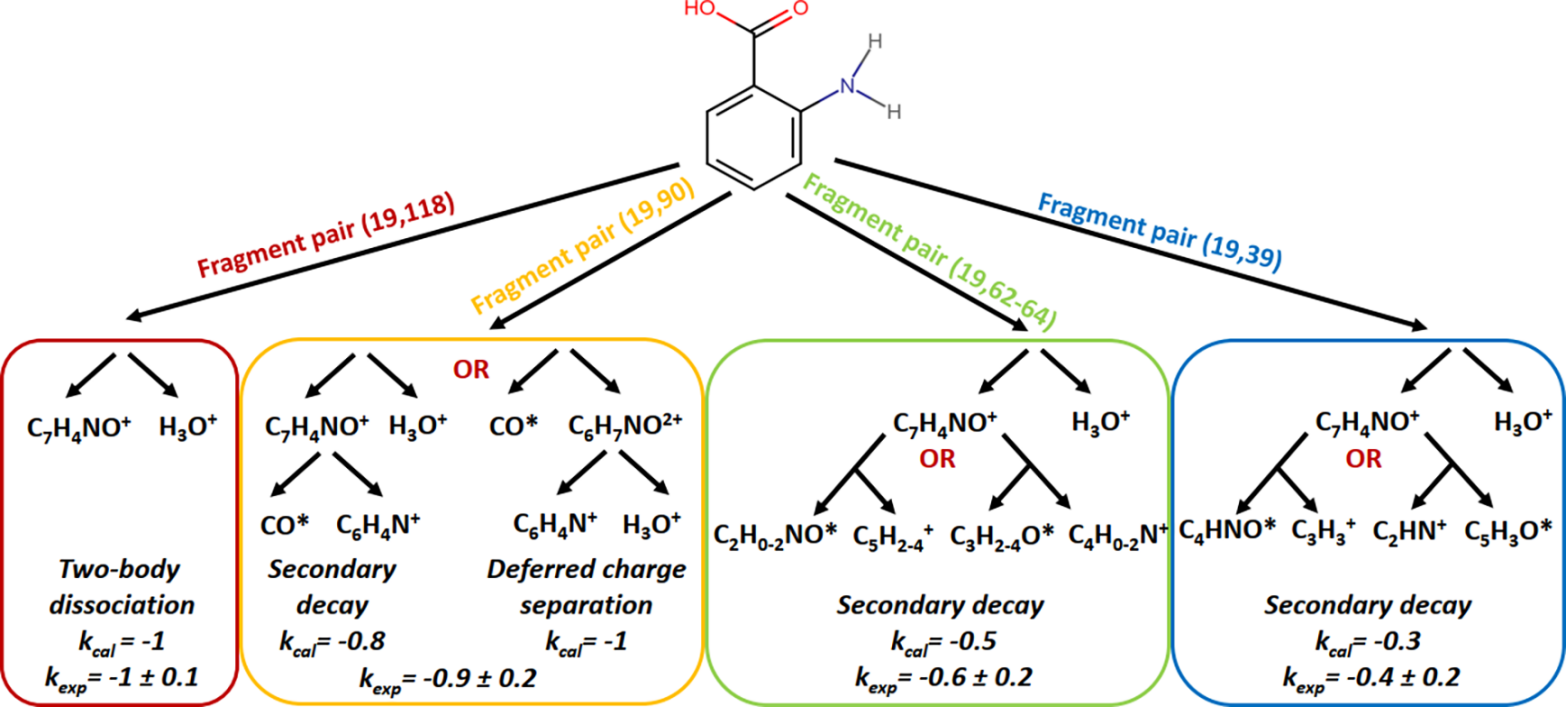
Suggested H_3_O^+^ involving fragmentation channels of ortho-ABA after C 1s photoionization. *k*_exp_ and *k*_cal_ indicate the slope of the corresponding pattern in the 2D map (Figure 4) and the modeled slope, respectively.

The most intense patterns in which one of the ions is H_3_O^+^ are (19, 118), (19, 90), (19, 62–64), and (19, 39). Especially pairs (19, 118) and (19, 90) have relatively well-defined slopes. We compare experimental *k*_exp_ and calculated *k*_cal_ slope values and suggest fragmentation mechanisms leading to these ion pairs in Figure 5. The coincident ion pair (19, 118) is self-evidently a two-body dissociation of the parent molecule (mass 137 u). The two-body dissociation produces a slope according to the ratio of charges of the two fragments, in this case *k*_cal_ = −1 as both are singly charged.^38^ The next coincident ion pair (19, 90) is produced via three-body dissociation, where undetected neutral fragment(s) have a total mass of 28 u. For this (19, 90) ion pair pattern, we find *k*_exp_ = −0.9 ± 0.2, and two possible mechanisms are considered. So-called deferred charge separation would produce *k*_cal_ = −1: first, a neutral CO is emitted and an intermediate fragment C_6_H_4_NH_2_OH^2+^ is formed, which then fragments to C_6_H_4_N^+^ and H_3_O^+^. Another alternative would be a secondary decay where H_3_O^+^ is separated first followed by fragmentation of the remaining singly charged ion in the second step. According to the simplified model of the momentum correlation, the slope would be a ratio of masses of the final singly charged fragment and the intermediate singly charged fragment from which it is produced, thus *k*_cal_ = 90/118 = −0.76.^38^ While deferred charge separation results in a slope closer to the experimental observation, the secondary decay slope is within the error bars, and these mechanisms can even be competing. The radial distribution of hydronium ions from the (19, 90) ion pair (see Figure S3c,d) is more widely spread over the detector than in the two-body dissociation. This is in agreement with the interpretation that the emission of the neutral fragment takes place first, giving some initial momentum to the further fragmenting ion. For the (19, 62–64) and (19, 39) ion pairs the statistics are not as good as for the pairs discussed above, but the rough estimates agree with the secondary decay model where H_3_O^+^ is emitted first and the singly charged 118 fragment dissociates further. This resembles the scheme found by Kling et al. in ethanol, where the transfer of the second hydrogen is concerted by the breakage of the C–O bond releasing H_3_O^+^.^5^

The ortho versus meta isomer-dependency and observed ion pairs invoke an interesting question of what would be the role of the two possible rotamers of ortho-ABA (Figure 1) in the production of H_3_O^+^. According to our calculations (performed at the CCSD(T)-level, see the Supporting Information for further details), the rotamer 1 has about 0.12 eV lower total energy than the rotamer 2, in agreement with previous calculations by Maciel et al.^39^ In accordance with the Boltzmann distribution analysis, the rotamer 1 constitutes approximately 98.5% of the population at 333 K. The higher energy rotamer 2 has the hydroxyl group closer to the amine group in the ground state, which could be expected to favor H_3_O^+^ production. However, given the small population of the rotamer 2, it is unlikely to be solely responsible for the H_3_O^+^ yield. Nuclear dynamics in the core-ionized and dicationic Auger final states are more likely involved, independent of the rotational configuration of the ortho-ABA isomer. Further work is needed to understand how the different rotamers contribute to the production of H_3_O^+^.

## Conclusions

In conclusion, we have observed H_3_O^+^ production in core-ionized aminobenzoic acid and studied specific fragmentation pathways for its production in ortho-ABA. The H_3_O^+^ creation takes place after at least two hydrogen atoms or ions migrate within the molecule to an oxygen site. This process is much more likely in ortho-ABA than in meta-ABA, which indicates that the amine group plays a major role as a donor. Due to this sensitivity to the molecular structure, in the future it would be interesting to study the intramolecular hydrogen transfer processes distinguishing the two energetically close-lying rotamers of ortho-ABA. Overall, ABA with its several isomeric forms is a compelling option for experimental and theoretical studies of detailed hydrogen migration dynamics using, for example, time- and momentum-resolved photoelectron diffraction.^40^ Multiple intramolecular hydrogen transfer processes may be more ubiquitous than previously thought in organic molecules, and especially the strong evidence for H-transfer dynamics between amine and carboxyl groups gives an interesting prospect when studying fragmentation of amino acids and peptides. Given the importance of H_3_O^+^ formation and H-transfer mechanisms in different environments ranging from biological to interstellar matter, we hope that this work inspires further experimental and theoretical work toward understanding the exact dynamics of these processes.

## Supporting Information Available

Details of the theoretical calculations, optimized geometries of meta-ABA and two rotamers of ortho-ABA; comparison of TOF mass spectra in coincidence with C 1s, N 1s, and O 1s photoelectrons for ortho-ABA; radial distribution maps of ortho-ABA ions; and list of *m*/*z* values of observed fragments and their possible molecular formula (PDF)
