# Supplementary material for: Forming Bonds While Breaking Old Ones: Isomer-Dependent Formation of H3O+ from Aminobenzoic Acid During X-ray-Induced Fragmentation
Source: J Phys Chem A. 2023 Feb 7;127(6):1395–401. doi: 10.1021/acs.jpca.2c06869 (PMC9940210; doi:10.1021/acs.jpca.2c06869)
Supplement: Supplementary file 1 — jp2c06869_si_001.pdf [file jp2c06869_si_001.pdf]

Supplementary Information for Publication "Forming Bonds While Breaking Old Ones: Isomer-dependent Formation of H_3O^+ from Aminobenzoic Acid During X-ray Induced Fragmentation"

Abdul Rahman Abid,[†,‡] Onni Veteläinen,[†] Nacer Boudjemia,[†] Eetu Pelimanni,[†] Antti Kivimäki,[†,¶] Matti Alatalo,[†] Marko Huttula,[†] Olle Björneholm,[‡] and Minna Patanen[*,†]

†*Nano and Molecular Systems Research Unit, Faculty of Science, P.O. Box 3000, 90014 University of Oulu, Finland*

‡*Molecular and Condensed Matter Physics, Uppsala University, Box 516, 75120 Uppsala, Sweden*

¶*MAX IV Laboratory, Lund University, P.O. Box 118, 22100 Lund, Sweden*

E-mail: minna.patanen@oulu.fi

Theoretical Calculations

Equilibrium ground state geometries of neutral ortho-ABA and meta-ABA were optimised using GAMESS *ab initio* quantum chemistry package (version 2019 R1)[1], at Restricted Hartree-Fock level[2] using 2nd order Møller-Plesset perturbation theory (MP2)[3–5]. Correlation-

consistent polarized core valence triple zeta (cc-pCVTZ) basis sets were used for all elements[6,7]. The optimised geometries are presented in Tables S1, S2, and S3 in Supplementary information for rotamer 1 of ortho-ABA, rotamer 2 of ortho-ABA, and meta-ABA, respectively. At the optimised geometries, single point energy calculations were performed with a Coupled-Cluster (CC) method, with a full treatment up to double excitations while triple excitations were taken into account as non-iterative correction (CC-SD(T))[8], and the converged energies were -12937.05883877595 eV (ortho-ABA rotamer 1), -12936.93811337704 eV (ortho-ABA rotamer 2), and -12936.92759052768 eV (meta-ABA).

Equilibrium ground state geometries for neutral ortho-ABA and meta-ABA

The optimised geometries are presented in Tables S1, S2, and S3 for rotamer 1 of ortho-ABA, rotamer 2 of ortho-ABA, and meta-ABA, respectively.

Table S1: Equilibrium geometry (in Å) of the rotamer 1 of ortho-ABA.

Atom	Charge	X	Y	Z
O	8.0	2.2477959344	1.5103279321	-0.1146993258
O	8.0	2.4883552252	-0.7147394060	-0.0723980300
N	7.0	0.2557256055	-2.2172626054	-0.3014640704
C	6.0	0.2763123394	0.2151649688	-0.0648649977
C	6.0	-0.4005637610	-1.0233237607	-0.1254104928
C	6.0	-0.4513013976	1.4091996816	0.0425868367
C	6.0	-1.8040461424	-1.0041036109	-0.0689615877
C	6.0	-1.8332146550	1.4019850876	0.0977667114
C	6.0	-2.5083095768	0.1808931900	0.0436151225
C	6.0	1.7465686777	0.2533064889	-0.0852281271
H	1.0	0.0930409112	2.3402034640	0.0884770767
H	1.0	-2.3360700086	-1.9459892870	-0.1214980939
H	1.0	-2.3799843098	2.3296132336	0.1842796609
H	1.0	-3.5889560291	0.1530778040	0.0863732855
H	1.0	1.2428427342	-2.2024999578	-0.0967017956
H	1.0	-0.2487570881	-3.0389935843	-0.0196367834
H	1.0	3.2084615406	1.3899403615	-0.1158353896

Table S2: Equilibrium geometry (in Å) of the rotamer 2 of ortho-ABA.

Atom	Charge	X	Y	Z
O	8.0	2.3509550622	1.4106617295	-0.1810116831
O	8.0	2.4692493978	-0.8217679378	-0.0612955274
N	7.0	0.2277282336	-2.2709102372	-0.3363488195
C	6.0	0.3018265233	0.1844264192	-0.0766185578
C	6.0	-0.3844954604	-1.0472360830	-0.1373774262
C	6.0	-0.4201498304	1.3810099046	0.0389480550
C	6.0	-1.7875219849	-1.0108285123	-0.0708900038
C	6.0	-1.8005016795	1.3909905729	0.1062993742
C	6.0	-2.4858806858	0.1765460209	0.0524684230
C	6.0	1.7716872792	0.3502809353	-0.1178957603
H	1.0	0.1482960725	2.2992145045	0.0779932367
H	1.0	-2.3277111101	-1.9481139761	-0.1256254788
H	1.0	-2.3364778171	2.3241046695	0.2007676276
H	1.0	-3.5661867876	0.1538736527	0.1038541274
H	1.0	1.1994906620	-2.3235917584	-0.0868679289
H	1.0	-0.3189942170	-3.0602337766	-0.0377082542
H	1.0	3.3989563425	-0.5507761279	-0.0918814040

Table S3: Equilibrium geometry (in Å) of meta-ABA.

Atom	Charge	X	Y	Z
O	8.0	2.2869965169	1.4768173266	-0.0205492042
O	8.0	2.4285886384	-0.7554353248	-0.2489471688
N	7.0	-2.4406825725	-2.2521533417	-0.3242032598
C	6.0	0.2660553668	0.2564104954	-0.0873504687
C	6.0	-0.3872097519	-0.9699019199	-0.1946673494
C	6.0	-0.4568215686	1.4419873451	0.0575718740
C	6.0	-1.7801654202	-1.0360887113	-0.1548451272
C	6.0	-1.8455318639	1.3758976687	0.0925861307
C	6.0	-2.5027825108	0.1554082355	-0.0139729455
C	6.0	1.7468676969	0.2391570312	-0.1303491689
H	1.0	0.0600100891	2.3852279902	0.1419230477
H	1.0	-2.4256696636	2.2817664540	0.2041346148
H	1.0	-3.5851138726	0.1202007462	0.0087322370
H	1.0	-1.8909789075	-3.0535217242	-0.0585575481
H	1.0	-3.3533731580	-2.2846459814	0.1005387565
H	1.0	3.2437719989	1.3340271475	-0.0610740437
H	1.0	0.2091989833	-1.8658934372	-0.3091303761

TOF Mass Spectra

Figure S1 shows a comparison of ortho-ABA TOF spectra recorded in coincidence with C 1s, N 1s, and O 1s photoelectrons ionized with photon energies of 350, 450, and 600 eV, respectively. The spectra were normalized by the integrated intensity. The mass spectra at all edges look overall similar except for increased yield of m/z = 16 and 17 in O 1s mass spectrum compared to C 1s and N 1s. These changes in O^+ and OH^+ yields are due to the ionization of residual water. The inset reveals that there is some site-specificity in the yield of H_3O^+.

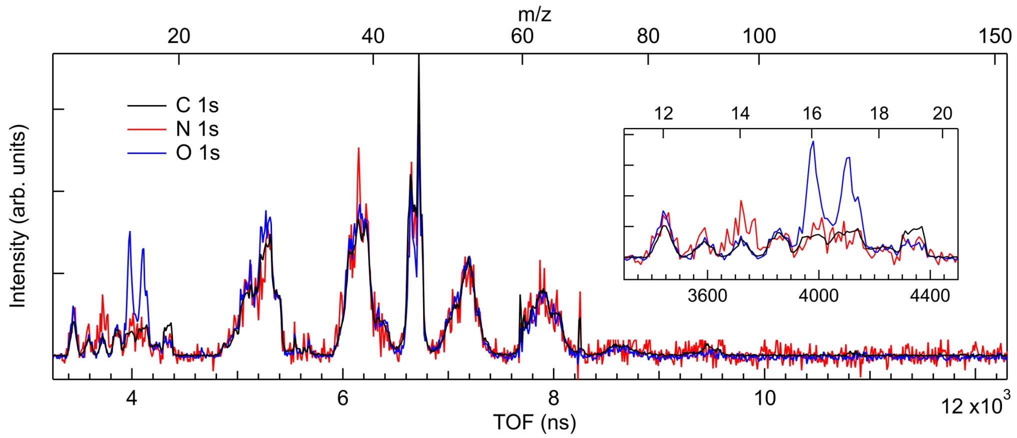

Figure S1: Comparison between TOF spectra of ortho-ABA recorded in coincidence with different core-level photoelectrons (C 1s (black line), N 1s (red line), and O 1s (blue line)). The top axis indicates the corresponding m/z range. Inset: enlarged TOF spectra from 3300-4500 ns, i.e. m/z ≈ 11 to 20.

Radial distribution maps of C 1s coincident ions of ortho-ABA

Figure S2 presents radial distribution maps, i.e. the distance of the ion's hit position from the center of the detector as a function of ion's TOF. Ions with no or very little kinetic energy arrive at the center of the detector (close to 0 mm) and form a confined spot in Fig. S2, for example doubly charged fragments m/z = 45.6, 56.6, 60, and 68.9 at 6720, 7682, 7714, 8249 ns, respectively. Ions which gained kinetic energy in the fragmentation process can arrive also at larger distances from the center. In some cases their hit position and TOF are clearly correlated and they form an arc (e.g. 19 u ion around 4340 ns as highlighted in Fig. S3), meaning that the radius of the ion's hit position depends on its original emission angle from the TOF spectrometer's axis. When an arc is not visible, the ion's kinetic energy can be very small or it is emitted in a sequential process where changes in momenta fade such correlation.

According to the PEPIPICO map shown in figure 3 of the main article, there is a small probability to produce a coincident ion pair (29, 90), which can be seen as a competing

process for the formation of the ion pair (19, 90): emission of COH^+ with neutral(s) adding up to 18 u (H_2O) vs. production of H_3O^+ with neutral(s) adding up to 28 u (CO). Coincident ion pair analysis shows that the latter process would be twice as abundant as the former, but it is possible that the COH^+ decays further by emission of H^+ which we unfortunately cannot observe due to the noise from the high voltage pulse masking the low m/z region of the TOF spectrum.

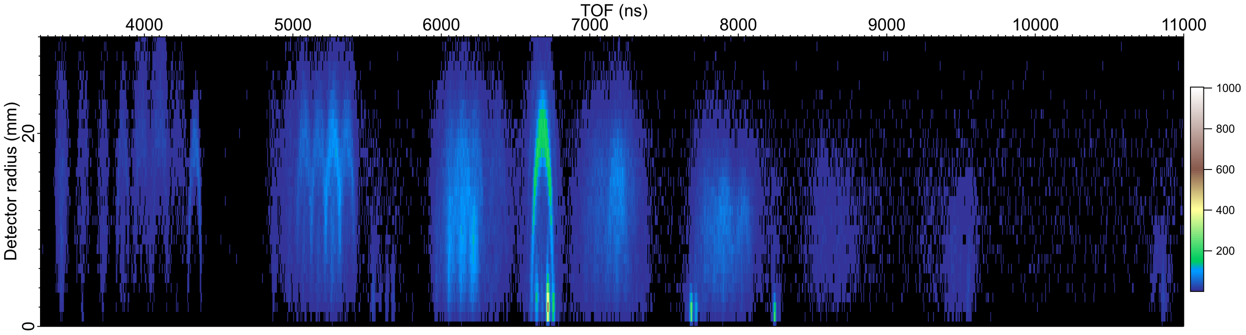

Figure S2: Radial distribution map of fragments of the ortho-ABA.

Fragments

Table S4 shows the detailed breakdown of the C 1s coincident mass spectrum of ortho-ABA with possible singly and doubly charged fragments. The core-ionization of ortho-ABA produces fragments ranging from m/z = 12 to 118. Due to the noise induced by the high voltage extraction pulse, we cannot detect ions below m/z = 10.

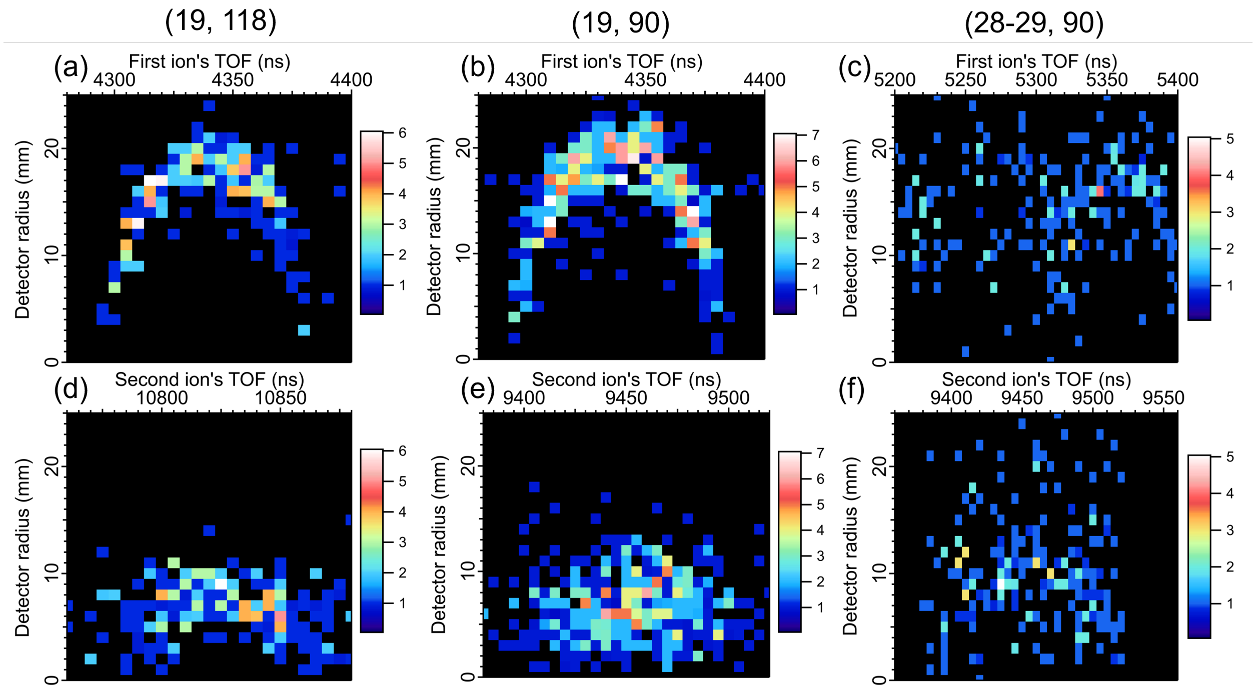

Figure S3: Radial distribution maps of fragments for coincident ion pairs (19, 118), (19, 90) and (28-29, 90). The upper maps are for the lighter ion and lower maps for the heavier ion of coincident pairs. For detector radius axis, zero is the center of the detector.

Table S4: Detailed breakdown of TOF coincident mass spectrum of ortho-ABA.

**Mass**	**Fragment**	**Mass**	**Fragment**	**Mass**	**Fragment**
12	C^+	31	HNO^+, H_3CO^+	61	$CH_3NO_2^+$
13	CH^+	32	H_2NO^+, O_2^+	63	$C_5H_3^+$, C_4HN^+
14	N^+	36	C_3^+	65	$C_5H_5^+$, $C_4H_3N^+$
15	NH^+	37	C_3H^+	68.8	$C_3H_3NO^+$, $C_7H_7NO_2^{2+}$
16	O^+, NH_2^+	38	$C_3H_2^+$, C_2N^+, $C_6H_4^{2+}$	72-76	$C_6H_{0-4}^+$
17	OH^+, NH_3^+	39	C_2HN^+, $C_3H_3^+$	90	$C_6H_4N^+$
18	H_2O^+	41	$C_2H_3N^+$	92	$C_6H_6N^+$
19	H_3O^+	45	CHO_2^+	118	$C_7H_4NO^+$
24	C_2^+	45.6	$C_6H_5N^{2+}$		
25	C_2H^+	50	$C_4H_2^+$, C_3N^+		
26	$C_2H_2^+$	52	$C_4H_4^+$, $C_3H_2N^+$		
28	CO^+, CH_2N^+	59.6	$C_7H_5NO^{2+}$		
29	CHO^+, CH_3N^+	60	${C_7H_4O_2}^{2+}$		

References

(1) Barca, G. M. et al. Recent developments in the general atomic and molecular electronic structure system. Journal of Chemical Physics **2020**, 152, 154102.

(2) Roothaan, C. C. New developments in molecular orbital theory. Reviews of Modern Physics **1951**, 23, 69–89.

(3) Pople, J. A.; Binkley, J. S.; Seeger, R. Theoretical models incorporating electron correlation. International Journal of Quantum Chemistry **1976**, 10, 1–19.

(4) Frisch, M. J.; Head-Gordon, M.; Pople, J. A. A direct MP2 gradient method. Chemical Physics Letters **1990**, 166, 275–280.

(5) Aikens, C. M.; Webb, S. P.; Bell, R. L.; Fletcher, G. D.; Schmidt, M. W.; Gordon, M. S. A derivation of the frozen-orbital unrestricted open-shell and restricted closed-shell second-order perturbation theory analytic gradient expressions. Theoretical Chemistry Accounts **2003**, 110, 233–253.

(6) Dunning, T. H. Gaussian basis sets for use in correlated molecular calculations. I. The atoms boron through neon and hydrogen. The Journal of Chemical Physics **1989**, 90, 1007–1023.

(7) Woon, D. E.; Dunning, T. H. Gaussian basis sets for use in correlated molecular calculations. V. Core-valence basis sets for boron through neon. The Journal of Chemical Physics **1995**, 103, 4572–4585.

(8) Raghavachari, K.; Trucks, G. W.; Pople, J. A.; Head-Gordon, M. A fifth-order perturbation comparison of electron correlation theories. Chemical Physics Letters **1989**, 157, 479–483.
